# Above-ground carbon stocks and its functional relationship with tree species diversity: the case of Kakamega and North Nandi Forests, Kenya

**DOI:** 10.1038/s41598-023-47871-6

**Published:** 2023-11-27

**Authors:** Ouko Amose Obonyo, Humphrey Agevi, Mugatsia Harrison Tsingalia

**Affiliations:** 1 Department of Biological Sciences, Masinde Muliro University of Science and Technology (MMUST), P.O. Box 190-50100, Kakamega, Kenya; 2 Institute of Indigenous Knowledge, Cultural Studies and Climate Change, Masinde Muliro University of Science and Technology (MMUST), P.O. Box 190-50100, Kakamega, Kenya

**Keywords:** Ecology, Environmental sciences

## Abstract

Estimating aboveground carbon (AGC) dynamics and tree diversity functionality relationships is critical in understanding the role of vegetation in implementing climate change mitigation strategies and promoting sustainable forest management. This study aimed to evaluate AGC stocks and their functional relationship with tree species diversity in Kakamega and North Nandi Forests, Kenya. A nested approach was adopted in sampling aboveground vegetation for biomass estimation in least disturbed, transformed, and disturbed sites. Tree biomass was estimated using an allometric equation based on tree diameter, tree height, and wood density. The biomass was then converted to carbon stocks using the carbon conversion factor. One-way ANOVA was used to determine the variation in carbon and tree diversity between forests and forest types. The correlation between tree diversity and AGC was evaluated. It was established that Kakamega Forest had the highest AGC (157.93 ± 26.91*tha*^−1^). The least disturbed areas had the highest AGC (65.96 ± 8.56*tha*^−1^). Additionally, Shannon diversity revealed a higher tree species diversity in Kakamega Forest (H′ = 1.82 ± 0.95). There was a significant positive correlation between AGC and tree species diversity (r = 0.62, p < 0.05). Kakamega and North Nandi forests vary in their AGC, and that tree species diversity positively influences the AGC of the two forests.

## Introduction

Climate change remains the biggest global concern in the twenty-first century because of its potential to adversely affect different biophysical environments^1,2^. Some of the adverse effects of climate change include biodiversity extinctions, reduced agricultural productivity, erratic weather patterns that may lead to droughts, floods, etc. and ultimately food insecurity; outbreaks and prevalence of emerging diseases, among others. Studies have proven that forests play a pivotal role in mitigating the negative impacts of climate change^3^.

Tropical forests are among the richest ecosystems in the world. They are thus important in the global carbon sink and cycle since they store large amounts of total terrestrial organic carbon through the exchange of carbon dioxide (CO2) with the atmosphere^4,5^. However, they are also habitats with the highest rate of degradation attributed to anthropogenic influences^6^. Primary attention has always been given to forests, which account for 45% of terrestrial carbon stocks and are responsible for 17% of annual radiative forcing through deforestation^3,7^. Nonetheless, the potential for carbon sequestration in the tropical rainforests is directly linked to tree species diversity^8^. The complex connection between tree species diversity and carbon stock has received particular emphasis over the last decades^9,10^ as they help to optimize the most environmental benefits of carbon storage and biodiversity conservation in the forest ecosystems.

High species richness and abundance contribute to high diversity which can significantly enhance ecosystem resilience and stability thereby promoting ecosystem health, primary production, biomass accumulation and eventually carbon sequestration potential below and aboveground forest ecosystems^11^. A clear understanding of how diversity affect ecosystem function is important as it provides direct strategies for the conservation and restoration of threatened natural ecosystems^12^. This presents an urgent need to estimate the level of tree species diversity and the corresponding carbon stocks stored in the existing forests^12^.

Measuring and managing ecosystems like forests based on their carbon stocks estimates, particularly under the umbrella of Reducing Emissions from Deforestation and Degradation (REDD +) posse greater benefit to biodiversity research and conservation^13,14^. References^12,15^ affirm that systematic assessment of carbon stocks contributes to the transparent analysis of complex and often contradictory science on forest carbon dynamics. According to Ref.^15^, productivity is the most common measure of ecosystem function and carbon storage potentials within most of the forest ecosystems. Given the difficulty in measuring productivity, standing biomass is frequently used as a proxy measure for ecosystem function.

Globally, forest ecosystems contain 85–90% of the total vegetation biomass^16^. These ecosystems however, suffer degradation and disturbances that hamper the forest ecosystem health and impairs their ability to effectively sequester carbon^17^. Major degradation drivers in forest ecosystems are anthropogenic and includes clearance of forests for large scale agriculture, urbanization, human settlement, commercial activities, social and recreational purposes, and conversion to pasture lands among others^18^. Reference^18^ further explained that natural causes such as climate change, wild forest fires, avalanches, volcanic eruptions and earthquakes cause degradation, but are less frequent than anthropogenic causes and that nature can easily balance them. The extent of these challenges facing forest ecosystems are global^19^.

Kenya’s tropical forests (Kakamega and North Nandi) are not exception to challenges facing other forests globally^20,21^. For instance, Ref.^22^ reported a cumulative forest land cover loss of 31% in Kakamega-Nandi forest between 1972 and 2001. The cumulative effects of these challenges, for instance, degradation, contribute to other collateral effects such as severe changes in the climatic patterns leading to droughts/flooding among others which further impact forest ecosystems, resulting to a reduction of their carbon sinks capacity^17^. Other long-term effects include reduced forest cover, increased degradation and fragmentation, and inability of forest ecosystems to effectively offer ecosystem goods and services such as food, buffering against harsh climatic events, flood control, habitat provision, water purification, among others.

The call for urgent action to avert climate change and its negative effects on the environment is real^23^. This study therefore assesses the spatial changes in Above Ground Carbon (AGC) stocks and the influence of tree species diversity in Kakamega and Nandi Forest ecosystems. The findings of this study provides a database that can be utilized by governments and various environmental agencies to inform sound management of forests locally and globally. This will not only foster mitigation of the negative effects of the global climate changes but also help in assessing the vulnerability of forest-adjacent communities to climate change in order to promote sustainable development and ecological stability.

## Materials and methods

### Study area

**Figure 1:**
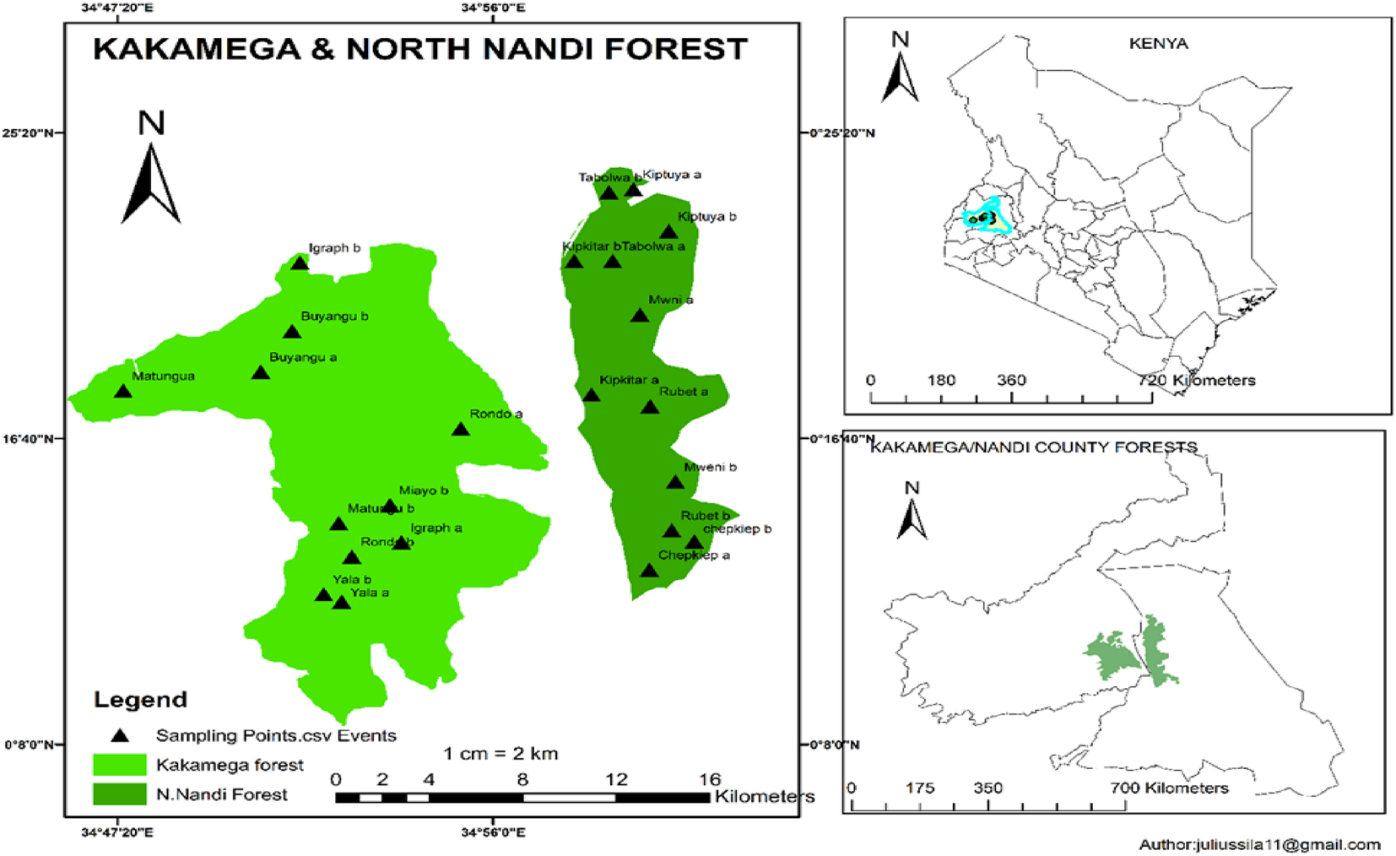
Study area, Kakamega and North Nandi Forests (Source: Author).

This study was done in the two tropical rain forests in western Kenya—the Kakamega, and North Nandi Forest (Fig. 1). Kakamega tropical rainforest is the only remnant of Guinea-Congolian forest in Kenya and together with the South and North Nandi Forest form the Kakamega-Nandi forest ecosystem^24^.

The Kakamega Forest is located in Kakamega county within the global position of between longitude 34º 40’ E and 34º 57′ 30’’ E; latitude 0º 15″ S and 0° 21′ S; and an altitude range between 1250 and 2000 m above the sea level^25^. The forest ecosystem experiences warm and wet climate with a bimodal rainfall. Short rain is experienced between July and October while long rain occurs between March and June. This cumulatively results to annual rainfall of between 1500 and 2000 mm^26^. Though this forest is largely cited to cover 240 square kilometers (km^2^), Ref.^27^ reported that this forest covered approximately 238 km^2^ by the year 2003, comprising of a national reserve and two nature reserves. The forest contains a variety of vegetation types, notably grasslands, bushlands, secondary and near-natural forests. In further description, Ref.^28^ reported that Kakamega Forest vegetation comprises of the secondary forest in diverse stages of succession, disturbed primary forest, mixed exotic and native plantation, and human-made and natural glades. Nevertheless, Ref.^27–29^ described the natural forest in Kakamega forest to constitute the primary forests within this tropical rain forest. The plantation forests, which were created from 1937 to 2005^28^ have a mix of indigenous and alien species. Nevertheless, Ref.^27^ affirmed that approximately 50% of the forest is natural forest. Because of succession, the dominance by tree species varies over time. For instance, ^28^ reported that *Funtumia africana, Croton megalocarpus, Antiaris toxicaria, Celtis mildebradii, and Ficus exasperate* are the most dominant species particularly in the natural forest. However, Ref.^30^ reported *Olea welwitschia* as the most dominant species in Kakamega forest. Nonetheless, Area around this forest is densely populated with a population density of 618 persons per km^2^^31^.

The North Nandi Forest is located in Nandi County. It has an area of about 10,501 ha^24,32^. The fores lies between longitude 34° 51′ 0" E and 35˚ 10′ 0" E; between latitude 0° 12′ 30" N and 0° 28′ 20″ N; and at an altitude range of between 1700 and 2500 m above the sea level^32^. The forest ecosystem experiences a bimodal rain pattern with long rain falling between March and June while short rain falls between September and October; cumulatively resulting to an average rainfall of between 1200 and 2000 mm per year^24^. The forest is drained by the rivers Clare, Kipkaren, Yala, Nonie, and Kingwal. North Nandi Forest is slightly higher in altitude and less diverse than Kakamega Forest area^20^. Just like Kakamega Forest, North Nandi forest is equally diverse with both near natural (primary forests), and man-made forests (plantations), plus disturbed natural forests^22^. The most dominant species of North Nandi forest include *Syzygium guineense, Vangueria madagascariensis, Croton macrostachyus, and Ehretia cymosa*^22^. Though reported as less diverse compared to Kakamega forest, Ref.^22^ indicated that North Nandi forest has unique tree species such as *Meyna tetraphylla*, and *Chrysophyllum viridifolium*, and other shrubs and herbs not found even in the species diverse Kakamega forest.

Forest-adjacent communities in both Kakamega and North Nandi Forest are small-scale mixed farmers who are dependent on the forests for their livelihoods^33–35^. These two forest were chosen for the study because of their differences in altitude and probably management regimes despite other factors remaining more or less similar.

### Data collection

Preceding the data collection was a reconnaissance study of the two study forests, in which individual forests were divided into four quarters based on forest boundary maps of each forest generated from Kenya forest Services archives. The geographic information system (ArcGIS) version 10.8 was used to obtain the Landsat images of the year 2020 from United States Geological Survey (USGS). In augmentation with aerial photographs, the land use/cover maps generated from Landsat images were used to demarcate the forest into 3 distinct forest types (least disturbed, plantations and disturbed areas). These were confirmed by the demarcation data of the least disturbed, plantations and disturbed sites as elaborated in each study forest’s management plan. Each of the forest quarters had at least all the three forest types. In this case, the least disturbed areas were areas of natural or mature old secondary forest whose management styles do not allow unauthorized access by the public (canopy cover not less than 70%). Due to reduced disturbances, the least disturbed areas should have high vegetation cover, more tree species of large trunks and aged species of trees. The reverse is true for the disturbed sites which constituted areas of natural forests that faced or were still facing perturbations (majorly anthropogenic such as over-browsing or natural such as floods) that do not allow full development of such areas to be dominated by woody trees (canopy cover not exceeding 30%). Plantations were forest areas which were either disturbed by natural or anthropogenic actions and have so far been restored to forest areas (majorly tree plantations of any type). Plantations were majorly identified from the respective forest inventory records from Kenya Forest service management plan. These forest types formed the treatments from which samples were drawn. This implies that each forest had 12 large quadrats of 50*50 m, totaling to 24 larger plots from which sampling was done.

Purposive sampling technique was used to position 50*50 m nested quadrats at least a one-kilometer interval in each treatment where sampling was done. To minimize sampling bias associated with the forest edge effects, the quadrats were established at least one-kilometer inwards from the forest edge. In each 50*50 m sampling plot, all the tree species were counted, identified to species level, and recorded. Trees with Dbh ≥ 5 cm were sampled for Dbh, height and wood density in the larger 50*50 m quadrat. Dbh was measured at 1.3 m above the ground using a Dbh tape. Tree heights were estimated using a suunto clinometer while wood density was obtained from the global wood density database, http://db.worldagroforestry.org/wd. Trees with Dbh < 5 cm were left out in DBH, height and wood density measurement because they constituted a very small fraction of AGB in the forests^36,37^. However, they were considered for biomass measurement as part of understory.

For the understory sampling, quadrats measuring 1*1 m were used. In this case and in each of the 50*50 m sampling plots in every treatment, 2 quadrats measuring 10*10 m were purposively established from which another set of 4 quadrats measuring 1*1 m were used for sampling the shrubs, herbs, and trees of Dbh less than 5 cm (understory). In each of the four 1*1 m quadrats, perpendicular lines were drawn on each side leading to four quarters each measuring 0.5*0.5 m. Two opposite facing quarters measuring 0.5*0.5 m were then picked in each 1*1 m for sampling whereby a knife was used to destructively cut off the trees of Dbh less than 5 cm, herbs, and shrubs at the center and four corners. The fresh weight (sample and sub-sample weights) of the understory samples were taken at the point of collection using an electronic weighing machine. The samples were then transported in a tight sample container to the MMUST laboratory for further processing (drying to a constant weight at a constant temperature of 80 °C for 72 h and their dry mass determined using an electronic weighing machine).

The understory data was fitted in the model {Total dry weight (kg/m^2^) = [TFW (kg) x SSDW (g)]/ [SSFW (g) × SA (m^2^)] described by Ref.^38^ to obtain understory biomass. In which case:

TFW- total fresh weight,

SSDW- Sub Sample Dry Weight,

SSFW- Sub Sample Fresh Weight, and

SA- Sample Area.

The Dbh, tree height and wood density were entered in an improved Chaves’ equation for the African moist tropical forests (W = F.ρD^2^H) described by Refs.^36,37^ to estimate the tree biomass of trees in each forest. In this case:

W - Tree biomass

F- Multiplicative coefficient

D- The predictor variable (DBH)

H- Tree height

ρ -wood specific gravity

The biomass of understory was added to the tree biomass to obtain the total aboveground biomass (TAB). AGB estimates obtained were converted to aboveground carbon (AGC) stocks using the default carbon fraction value of 0.47 from Intergovernmental Panel on Climate Change^39^. Using the excel, the Shannon Wienner Diversity Index (H´) was applied in calculating the tree diversity across the treatments:

$$\text{Shannon Index }\left({\text{H}}\right)= -\sum_{t=1}^{s}{p}_{t}{\text{ln}}{p}_{t},$$

where p is the proportion (n/N) of individuals of a particular species (n) divided by the total number of individuals (N), ln is the natural log, ‘Σ’ is the sum of the calculations, and ‘s’ is the number of species. Shannon Wiener diversity index considers species richness (total number of different species), tree abundance (total number of trees) and the relative species abundance or evenness (count of trees for each species).

### Data analysis

After cleaning the data, analysis was done using the Statistical Package for the Social Sciences (SPSS) version 25. Data was subjected to normality test (Shapiro–Wilk goodness-of-fit test) since the two sites were considered heterogeneous, and when found to be normally distributed, the variation in the carbon stock in the two sites were ascertained by performing one way analysis of variance (ANOVA). ANOVA was also used to assess the significant effect of tree species diversity on the above ground carbon stock. Pearson’s and Spearman's rho correlation tests were used depending on the normality of the data distribution to test the relationship between AGC and species diversity, AGC and DBH, tree species richness, and tree species abundance.

### Approval

This research was approved by the Directorate of graduate School (DPS) of Masinde Muliro University of Science and Technology (MMUST) as part of the Master of Science in Environmental Science. It was further approved by the National Commission of Science and Technology (NACOSTI) ref No: 631572.

## Results and discussions

### Aboveground carbon stocks

**Figure 2:**
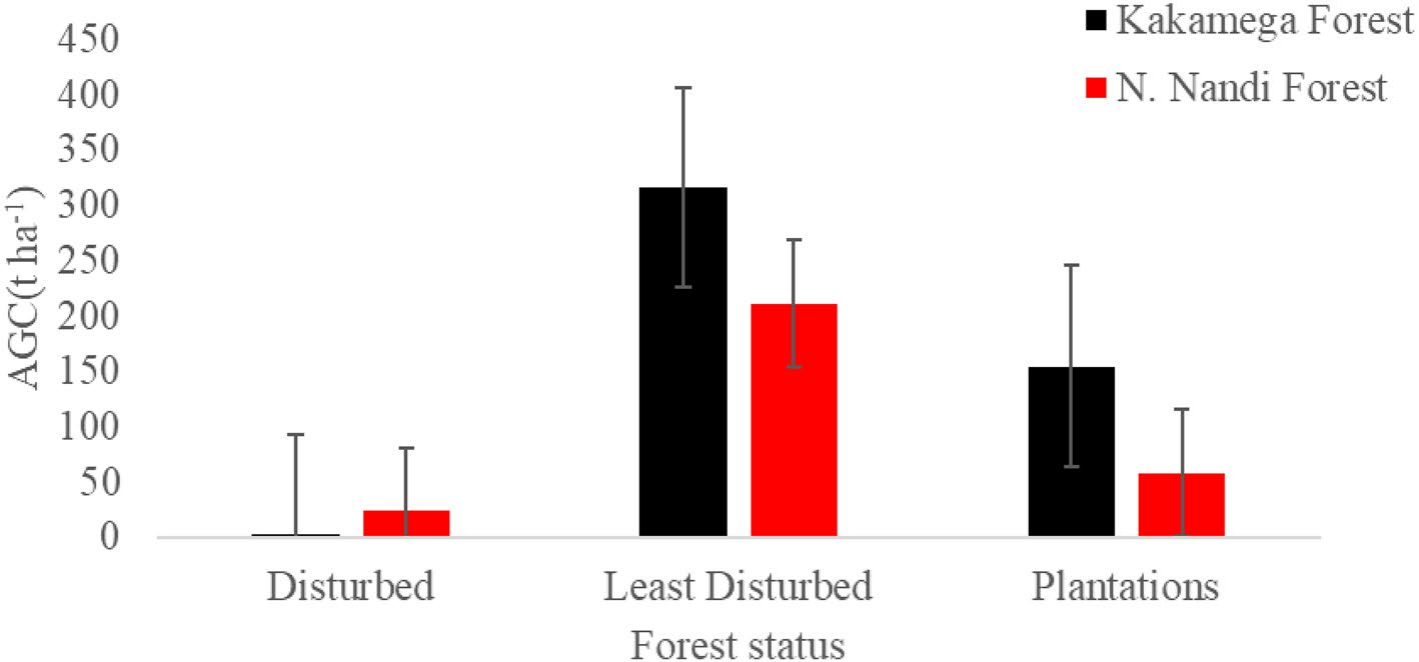
Carbon Stocks in disturbed, least disturbed and Plantation areas of North Nandi and Kakamega forests.

Kakamega Forest had the highest mean AGC (157.93 ± 156.91 t ha^−1^) while North Nandi Forest had (97.83 ± 99.89 t ha^−1^). Results of ANOVA revealed statistically significant spatial variation in AGC stock (F_(5,18)_ = 6.523; p = 0.001) among different forest types in Kakamega and North Nandi Forests. Least disturbed forest areas recorded the highest mean AGC (65.96 ± 38.56tha^−1^), followed by plantation/transformed sites (26.69 ± 17.43 tha^−1^), while disturbed forest type had (3.26 ± 7.11tha^−1^) (Fig. 2). In the Kakamega forest, AGC was highest in least disturbed sites (316.31 ± 215.64tha^−1^), followed by plantations (154.96 ± 54.99tha^−1^), and lastly disturbed sites (2.53 ± 1.77tha^−1^). The AGC variation among the forest types in the Kakamega Forest was significant at F_(2,9)_ = 5.966, p < 0.05. In the North Nandi Forest, AGC was highest in least disturbed sites (211.40 ± 40.82tha^−1^), followed by plantations (58.57 ± 46.06 t ha^−1^), and lastly disturbed sites (23.54 ± 39.85tha^−1^ (Fig. 2). Mean AGC among the forest types in the North Nandi Forest was significantly different at F_(2,9)_ = 22.274, p < 0.05.

### Tree species abundance, richness, and diversity

#### Tree species abundance

A total of (N = 1511) trees belonging to 100 different species were recorded in the two forests (Table 1). 27% (n = 27) of the total species were common to both Kakamega and North Nandi Forests while 28% (n = 28) of the total species were found only in North Nandi Forest. 45% (n = 45) were found only in Kakamega Forest. The most abundant species was *Cupressus lusitanica* with 26.9% (n = 407) of all individual trees while the least abundant species was *Vitex keniensis* with only a single individual tree (n = 1) representing 0.07%. North Nandi Forest had the highest number of trees sampled (n = 840: 55.6%), where *Cupressus lusitanica* was the most abundant species 38.6% (n = 324) while *Xymalos monospora* with (n = 1) individual (0.12%) was the least abundant species*.* In Kakamega Forest, the number of trees sampled comprised 44.4% (n = 671) with *Cupressus lusitanica* being the most dominant species at 12.4% (n = 83). *Milicia excelsa* was the least abundant at 0.05% (n = 1). In North Nandi Forest, plantation forests had the highest tree abundance 50.8% (n = 427) where *Cupressus lusitanica* was the most dominant species 75.9% (n = 324) while *Casearia battiscombei* the least dominant species at 0.23% (n = 1). The least disturbed forest was second highest in tree abundance at 41.5% (n = 348) of total tree sampled. *Syzygium guineense* was the most dominant species 16% (n = 56) while *Dracaena steudneri* was the least dominant species with only one individual (0.29%). Disturbed sites recorded the least tree abundance representing 7.7% (n = 65) with *Acacia nilotica* at 21% (n = 14) as the most dominant whereas *Maesa lanceolata* was the least dominant with only one individual (1.54%). In Kakamega Forest, the least disturbed sites had the highest tree abundance at 48% (n = 328) with *Funtumia africana* being the most abundant species representing 10.7% (n = 35) while the least abundant species being *Vangueria esculenta* 0.3% (n = 1). Plantation forest had the second highest tree abundance 44.9% (n = 301) with *Cupressus lusitanica* accounting for 27.6% (n = 83) making it the most abundant species while *Synsepalum afzelii* the least abundant 0.33% (n = 1). Disturbed sites had the least abundance of trees 6.3% (n = 42) with *Sesbania sesban* being the most abundant 32.9% (n = 13) while *Combretum collinum* being the least dominant species 2.4% (n = 1) (Table 1).

**Table 1:** Tree abundance, richness, and diversity between Kakamega and North Nandi Forests.

SITE	Forest status	N (%)	Richness	DBH (cm)			Diversity (H´)
				Min	Max	Mean	
Two Forests	Kakamega and North. Nandi	1511	100	5	130	27.28	
Kakamega Forest	All status combined	671 (44.4)	72	5	130	34.43	1.82 ± 0.95
North Nandi Forest	All status combined	840 (55.6)	55	5	121	26.02	1.24 ± 0.88
Kakamega Forest	Least disturbed	328 (48.9)	51	5	130	38.45	2.65 ± 0.45
	Plantations	301 (44.9)	49	5	110	34.33	1.84 ± 0.91
	Disturbed	42 (6.3)	12	6	11	11.2	0.98 ± 0.66
North Nandi Forest	Least disturbed	348 (41.5)	43	5	121	33.48	2.06 ± 0.45
	Plantations	427 (50.8)	9	5	100	19.31	0.32 ± 0.20
	Disturbed	65 (7.7)	18	5	108	26.9	1.36 ± 0.75

#### Tree species richness

Seventy-two (n = 72) species were recorded in the Kakamega Forest while North Nandi Forest recorded 55 tree species. Least disturbed sites of Kakamega Forest had more species (n = 51); followed by plantations (n = 49), while disturbed sites had the least richness (n = 12). In North Nandi Forest, the least disturbed sites had more species (n = 43); followed by disturbed sites (n = 18); while plantation areas had the least richness (n = 9) (Table 1).

#### Tree species diversity

**Figure 3:**
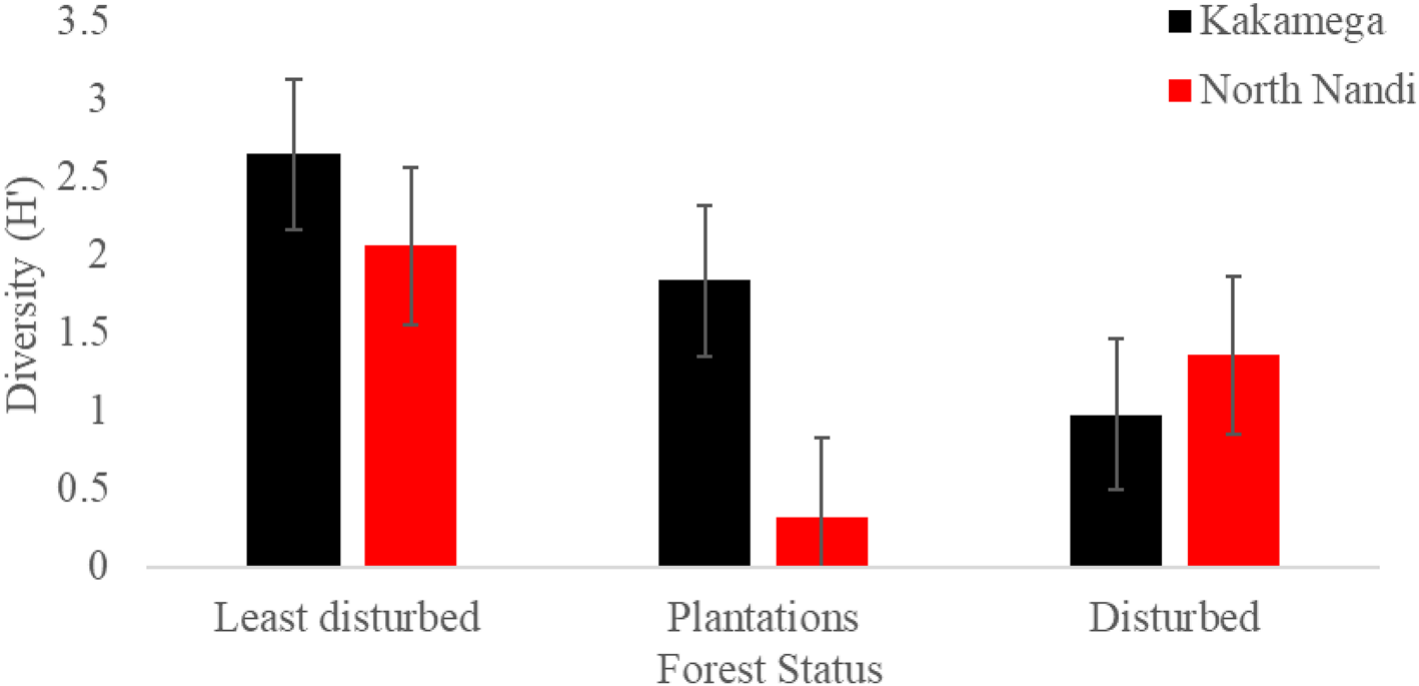
Mean Plant species diversity for Disturbed, least disturbed and Plantations of Kakamega and North Nandi Forest ecosystems.

Kakamega Forest recorded a higher tree species diversity (H´ = 1.82 ± 0.95) relative to North Nandi Forest’s (H´ = 1.24 ± 0.88). This difference was not significant at F = 2.406, *p* > 0.5. One way ANOVA revealed a statistically significant spatial variation in tree diversity among the different forest types in the two forests combined (F_(5,18)_ = 7.311, *p* < 0.05. Results of separate analysis on the basis of the forests revealed that in the Kakamega Forest, the least disturbed sites recorded the highest tree diversity (H´ = 2.65 ± 0.45) followed by plantation areas (H´ = 1.84 ± 0.91), and lastly disturbed areas (H´ = 0.98 ± 0.66). This variation was significant at F_(2,9)_ = 5.727, *p* < 0.05. In North Nandi Forest, least disturbed sites had the highest diversity (H´ = 2.06 ± 0.45), followed by disturbed sites (H´ = 1.36 ± 0.75), while plantation areas had the least diversity at H´ = 0.32 ± 0.20 (Fig. 3). This difference was statistically significant at F_(2,9)_ = 11.576, *p* < 0.05.

### Size class distribution

**Figure 4:**
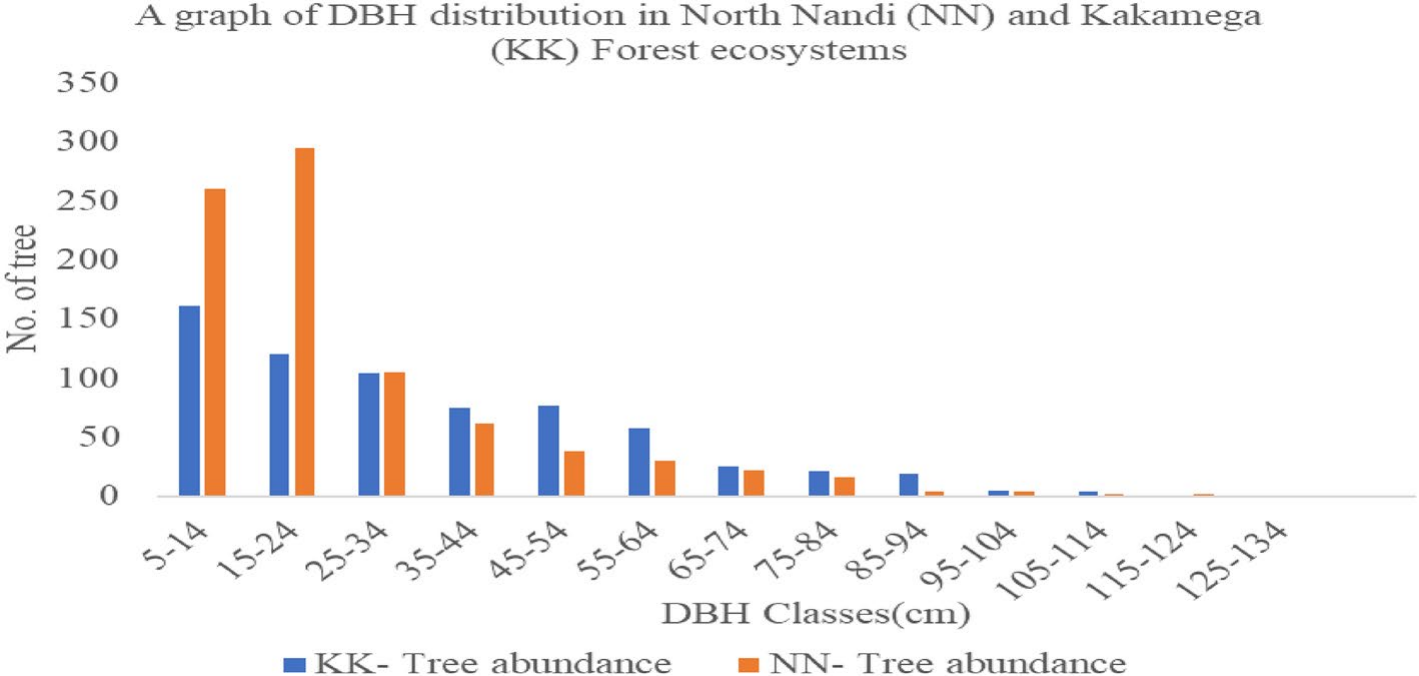
Size class distribution of trees in North Nandi and Kakamega Forest ecosystems.

The Dbh for the entire study area ranged between 5 and 130 cm with a mean Dbh of 27.28 cm. In Kakamega forest, least disturbed areas had a 5–130 cm Dbh range with an mean Dbh of 38.45 cm. Plantations site had a Dbh range of 5–110 cm with mean Dbh of 34.33 cm; while the disturbed sites had a Dbh range of between 6 and 48 cm with a mean Dbh of 11.2 cm (Table 1). The highest number of tree (n = 161) were found in the Dbh range of 5–14 cm while the least (n = 1) was found in the Dbh range of 115–125 cm and 125–134 cm respectively in Kakamega Forest (Fig. 4). In North Nandi Forest, least disturbed sites had a Dbh range of 5–120.5 cm with a mean Dbh of 33.48 cm; followed by plantation areas with a Dbh range of 5–100 cm and a mean Dbh of 19.31 cm; while disturbed sites had a Dbh range of 5–108 cm with a mean Dbh of 26.9 cm. The highest number (n = 295) of trees in North Nandi Forest were recorded in the Dbh range of 15–24 cm while the least number (1) was found in the Dbh range of 125–134 cm (Fig. 4).

### Above-ground carbon (AGC) stocks and tree species diversity relationships

The diversity of the two forests combined revealed a statistically significant positive relationship with AGC (r = 0.616, p < 0.05). Both Kakamega and North Nandi Forests independently revealed statistically significant positive correlation between AGC and tree species diversity (r = 0.665, p < 0.05; and r = 0.604, p < 0.05 respectively. Based on individual forests, there was a statistically significant strong positive correlation between AGC and tree species diversity in Kakamega least disturbed areas (r = 0.965, p < 0.05) followed by Kakamega disturbed sites (r = 0.603, p < 0.05); while Kakamega plantations recorded a weak positive correlation (r = 0.200, p < 0.05). In North Nandi Forest, the least disturbed areas had a statistically significant strong positive relationship (r = 0.800, p < 0.05) between AGC and tree species diversity; followed by North Nandi disturbed sites (r = 0.051, p < 0.05) and lastly a strong negative relationship between AGC and tree species diversity (r = -0.772 p < 0.05) in North Nandi Forest plantations.

#### AGC-Tree species abundance relationship

This study recoreded a statistically significant strong positive relationship between AGC and tree species abundance in the two forests combined (r = 0.679, p < 0.05). Kakamega Forest had a strong positive correlation between AGC and tree species abundance (r = 0.912, p < 0.05) whereas North Nandi Forest showed a moderate positive correlation (r = 0.378, p < 0.05). The least disturbed forest in Kakamega Forest showed a strong positive relationship between AGC and tree species abundance (r = 0.962, p < 0.05) followed by Kakamega plantations (r = 0.873, p < 0.05), while the disturbed areas in Kakamega forest had a weak negative relationship (r =  − 0.089, p < 0.05). In North Nandi Forest, the least disturbed forest areas had a statistically significant strong positive correlation (r = 0.800, p < 0.05) between AGC and tree abundance; followed by a weak negative correlation (r =  − 0.092, p = 0.05) in disturbed sites, while North Nandi Forest transformed recorded a strong negative correlation between AGB and tree species abundance (r =  − 0.738 and p < 0.05) (Table 2).

**Table 2:** The Correlation between AGC and tree species diversity, species richness, species abundance, and species richness. Significant values are in italic.

SITE	Forest status	Tree sp. Diversity (H´)	AGC Stocks (t ha^-1^)	Correlation variables			
		Mean ± std deviation	Mean ± std deviation	AGC & tree Sp. abundance	AGC & tree Sp. Richness	AGC & tree Sp. DBH	AGC and tree sp. biodiversity
2 Forests Combined	Kakamega & North Nandi			*r* = *0.679 p* = *0.01*	*r* = *0.856 p* = *0.01*	*r* = *0.915 p* = *0.01*	*r* = *0.616 p* = *0.01*
Kakamega Forest	all status combined	1.82 ± 0.95	157.93 ± 156.91	*r* = *0.912 p* = *0.01*	*r* = *0.78 p* = *0.01*	*r* = *0.909 p* = *0.01*	*r* = *0.665 p* = *0.05*
North Nandi Forest	all status combined	1.24 ± 0.88	97.83 ± 99.89	*r* = *0.378 p* = *0.05*	*r* = *0.806 p* = *0.01*	*r* = *0.828 p* = *0.01*	*r* = *0.604 p* = *0.05*
Kakamega Forest (analyzed per status as 1 forest)	Least Disturbed	2.65 ± 0.45	316.31 ± 215.64	*r* = *0.962 p* = *0.01*	*r* = *0.935 p* = *0.01*	*r* = *0.999 p* = *0.01*	*r* = *0.965 p* = *0.05*
	Plantations	1.84 ± 0.91	154.96 ± 54.99	*r* = *0.873 p* = *0.05*	*r* = *0.400 p* = *0.05*	*r* = *0.800 p* = *0.05*	*r* = *0.200 p* = *0.05*
	Disturbed	0.98 ± 0.66	2.53 ± 1.77	*r* = *− 0.089 p* = *0.05*	*r* = *0.344 p* = *0.05*	*r* = *0.021 p* = *0.05*	*r* = *0.603 p* = *0.05*
North Nandi Forest (analyzed per status as 1 forest)	Least Disturbed	2.06 ± 0.45	211.40 ± 40.82	*r* = *0.800 p* = *0.05*	*r* = *0.800 p* = *0.05*	*r* = *0.083 p* = *0.05*	*r* = *0.800 p* = *0.05*
	Plantations	0.32 ± 0.20	58.57 ± 46.06	*r* = *− 0.738 p* = *0.05*	*r* = *0.687 p* = *0.05*	*r* = *0.400 p* = *0.05*	*r* = *− 0.772 p* = *0.05*
	Disturbed	1.36 ± 0.75	23.54 ± 39.85	*r* = *− 0.092 p* = *0.05*	*r* = *− 0.200 p* = *0.05*	*r* = *1.00 p* = *0.01*	*r* = *0.051 p* = *0.05*

#### AGC-tree species richness relationships

There was a strong positive correlation between species richness and AGC (r = 0.85, p < 0.05) in the two forests combined. Statistically significant strong positive correlation was observed separately for North Nandi Forest (r = 0.806, p < 0.05) and Kakamega forest (r = 0.79, p < 0.05). Separate analysis of the forests based on the forest type revealed that Kakamega Forest had a statistically significant strong positive relationship between AGC and tree species richness in least disturbed areas (r = 0.935, p < 0.05), followed by a moderate positive relationship in both plantations and disturbed sites (r = 0.400, p < 0.05; and (r = 0.344, p < 0.05) respectively. In North Nandi Forest, least disturbed sites had a significant strong positive relationship between AGC and species richness (r = 0.800, p < 0.05); followed by plantations (r = 0.687, p < 0.05) while disturbed sites revealed a weak negative correlation between species richness and AGC (r = -0.200, p < 0.05) (Table 2).

#### AGC-Tree species DBH relationships

There was a strong significant positive correlation between AGC and Dbh for the teo study forests combined (r = 0.92 a p < 0.05). The strong positive correlation between Dbh and AGC was also recorded in both Kakamega Forest (r = 0.909, p < 0.05) and North Nandi Forest (r = 0.83; p < 0.05) respectively. A significantly strong positive correlation was recorded between AGC and Dbh in Kakamega least disturbed (r = 0.999 at p < 0.05) and plantation (r = 0.8 and p < 0.05); while Kakamega disturbed sites recorded a weak positive correlation between Dbh and AGC at r = 0.021, p < 0.05. In North Nandi disturbed sites, a perfect positive correlation (r = 1.00 at p < 0.05) was recorded. North Nandi plantations however revealed a moderate positive correlation between AGC and Dbh with a correlation coefficient of r = 0.400, p < 0.05; while a weak positive correlation between AGC and Dbh was recorded in North Nandi least disturbed areas at r = 0.083, p < 0.05) (Table 2).

## Discussion

Higher AGC stocks were recorded in the Kakamega Forest relative to North Nandi. The current figure of AGC in the Kakamega forest is above 0.2 tons ha^-1^ reported between the years 1987–2003 by Ref.^40^. The increase in carbon stock per hectare as revealed in this study could be attributed to several factors including enhanced Kenya government policy on reforestation to achieving the 10% forest cover by 2030, that has seen a significant embrace of on-farm and urban forestry which may have significantly reduced pressure on public forests by forest adjacent communities^33,41^. It could also be attributed to massive awareness campaigns by NGOs on the importance of planting more trees; that has significantly reduced the pressure on the Kenya’s gazetted forests by the local communities living adjacent to these forests. This finding agrees to several studies including that by Refs.^42–44^ in West Africa that all reported an improved conservation status and tree biomass accumulation in the forest system under the influence of participatory forest management focused on awareness creation led by the NGOs and local community.

The trend of high AGC stocks showed in least disturbed forest areas, followed by plantation areas, and lastly disturbed areas; and also reflected in both North Nandi and Kakamega forests when analyzed separately, could be attributed to the impact of forest disturbance/ management regime on carbon sequestration potential of forests^8^. In this study, the least disturbed areas were areas of natural or mature old secondary forest whose management styles do not allow unauthorized access of public forests by humans. Due to reduced disturbances, the least disturbed areas have high vegetation cover, more tree species of large trunks and aged species capable of sequestering huge amount of carbon relative to disturbed areas with low tree species abundance, little aboveground vegetation and consequently low carbon. This finding agrees with^45^’s finding in North America that forest disturbances from land use changes, wildfires among others continuously reduce the forest cover, and consequently reduce carbon storage potential by 11TgC (teragrams of carbon) annually in North America.

This study also agrees with a study in Ethiopia by Ref.^46^ assessing the carbon variation along different management regime which found a high AGC stock in the least disturbed areas relative to small carbon stock in highly disturbed areas. This finding agrees to the finding by Ref.^8^ in Southeast China, and Ref.^47^’s finding in tropical African forest which reported that tree species diversity was positively correlated with AGC. This finding also agrees with the finding by Ref.^48^ assessing forest cover dynamics, their drivers, and implications in western Kenyan forest ecosystems, which reported that management regime under the KFS and KWS enhance the forest protection against deforestation and other anthropogenic disturbances hence high species diversity and richness thus enhanced biomass carbon accumulation over time.These findings, however, contradict the finding by Ref.^49^ which found no correlation between species diversity and aboveground carbon in the sub-tropical forest of Eastern China. Furthermore, the high trends of carbon in least disturbed areas, followed by plantations and lastly disturbed sites in both Kakamega and North Nandi Forests could be attributed to differences in tree species diversity, richness and abundance. The species richness, abundance, and diversity were least in disturbed sites, medium in plantations and highest in least disturbed areas; and so was their AGC stocks. The highest abundance of trees were concentrated in DBH ranges of 15–4 cm. These were trees that held most of the estimated carbon. The concentration of biomass in a small number of trees has been previously observed in other moist forest ecosystems^50–52^. This, according to Ref.^13^ has clear implications for the development of rapid, low-cost forest monitoring protocols. Trees with a bigger DBH were fewer but stored substantial good amounts of biomass which translates into large quantities of stored carbon. This finding agrees with many findings including^49,53^. This finding, however, contradicts finding by Ref.^54^ which reported that Dbh had negligible and insignificant influence on aboveground carbon in tropical forests. High species richness and abundance contribute to high diversity and have the ability to significantly enhance ecosystem resilience and stability thereby promoting ecosystem health, primary production, biomass accumulation and eventually carbon sequestration potential of vegetation. As reported also by Ref.^11^ in their cross-review study evaluation the forestry systems that optimize carbon sequestration potential, a high tree species diversity enhances both ecosystem health and stability thus promoting both forest biomass and carbon above the ground. This study also revealed a strong positive correlation between tree species abundance and the AGB, and corespondingly AGC stocks. A higher tree abundance results in a high cumulative biomass of an ecosystem due to high primary productivity and this may explain the positive tree abundance and AGC relationship. This finding agrees with the finding in Southern China forest by Ref.^55^ and another study by Ref.^8^ that revealed a strong positive correlation between tree abundance, primary productivity, and biomass; which translate into the AGC stock. In all the sampled forest types, Dbh and AGC positively correlate.

## Conclusions and recommendations

### Conclusions

Kakamega and North Nandi forests vary in above ground carbon stocks, and that Kakamega forest is more stocked in terms of carbon than the North Nandi Forest. Least disturbed forest areas have relatively higher carbon storage potential compared to both plantations and disturbed site. This underscores the influence of management regime/forest disturbances, and species diversity on the forest development and functionality. Tree species diversity and Dbh positively impacts carbon stocks as shown in both Kakamega and North Nandi Forests. This underscores the time factor plus disturbances in influencing forest development and structural components and thus carbon storage potential. Kakamega forest was more diverse compared to North Nandi, however, least disturbed areas are the most diverse, followed by plantations and then disturbed sites. Again, this explains the role that disturbances play in destabilizing the species abundance and richness of a forest system and its riffle impact on forest functions such as climate change mitigation via carbon sequestration. This shows that the management regime and disturbance level significantly influence the carbon storage level of a forest ecosystem.

### Recommendations

1. Diversity of trees should be highly encouraged in forestry systems to enhance more carbon sequestration and storage above the ground. Mixed indigenous plantations should as well be encouraged and adopted in the forest ecosystems as opposed to pure stand plantations while at the same time protecting the existing least disturbed forest areas and or restoring disturbed forest ecosystems to the least disturbed or thicket nature/state.
2. Awareness creation and public involvement in every stage of forestry management should be enhanced to reduce pressure on public forests. Further studies should be done to investigate the below ground carbon stocks to fully understand the role these forest compartments play in forest carbon dynamics.

## Data Availability

The datasets generated during and/or analysed during the current study are available from the corresponding author on reasonable request.

## References

[1] Lin B, Ouyang X (2014). Analysis of energy-related CO_2_ (carbon dioxide) emissions and reduction potential in the Chinese non-metallic mineral products industry. Energy.

[2] Tozer L, Bulkeley H, Kiss B (2023). Nature for resilience? The politics of governing urban nature. Ann. Am. Assoc. Geogr..

[3] Ramachandra TV, Bharath S (2020). Carbon sequestration potential of the forest ecosystems in the Western Ghats, a global biodiversity hotspot. Nat. Resour. Res..

[4] Navarrete-Segueda A, Martínez-Ramos M, Ibarra-Manríquez G (2018). Variation of main terrestrial carbon stocks at the landscape-scale are shaped by soil in a tropical rainforest. Geoderma.

[5] Keller DP, Feng EY, Oschlies A (2014). Potential climate engineering effectiveness and side effects during a high carbon dioxide-emission scenario. Nat. Commun..

[6] Temgoua LF, Momo Solefack MC, Nguimdo Voufo V (2018). Spatial and temporal dynamic of land-cover/land-use and carbon stocks in Eastern Cameroon: A case study of the teaching and research forest of the University of Dschang. For. Sci. Technol..

[7] Birdsey R, Angeles-Perez G, Kurz WA (2013). Approaches to monitoring changes in carbon stocks for REDD+. Carbon Manag..

[8] Liu X, Trogisch S, He J-S (2018). Tree species richness increases ecosystem carbon storage in subtropical forests. Proc. R. Soc. B.

[9] Gebrewahid Y, Meressa E (2020). Tree species diversity and its relationship with carbon stock in the parkland agroforestry of Northern Ethiopia. Cogent. Biol..

[10] Van Con T, Thang NT, Khiem CC (2013). Relationship between aboveground biomass and measures of structure and species diversity in tropical forests of Vietnam. For. Ecol. Manag..

[11] Di Sacco A, Hardwick KA, Blakesley D (2021). Ten golden rules for reforestation to. optimize carbon sequestration, biodiversity recovery and livelihood benefits. Glob. Change Biol..

[12] Mensah S, Veldtman R, Du Toit B (2016). Aboveground biomass and carbon in a South African mistbelt forest and the relationships with tree species diversity and forest structures. Forests.

[13] McNicol IM, Ryan CM, Dexter KG (2018). Aboveground carbon storage and its links to stand structure, tree diversity and floristic composition in south-eastern Tanzania. Ecosystems.

[14] Bucki M, Cuypers D, Mayaux P (2012). Assessing REDD+ performance of countries with low monitoring capacities: The matrix approach. Environ. Res. Lett..

[15] Petrokofsky G, Kanamaru H, Achard F (2012). Comparison of methods for measuring and assessing carbon stocks and carbon stock changes in terrestrial carbon pools. How do the accuracy and precision of current methods compare? A systematic review protocol. Environ. Evid..

[16] Guo Z, Hu H, Li P (2013). Spatio-temporal changes in biomass carbon sinks in China’s forests from 1977 to 2008. Sci. China Life Sci..

[17] Garrard SL, Beaumont NJ (2014). The effect of ocean acidification on carbon storage and sequestration in seagrass beds; a global and UK context. Mar. Pollut. Bull..

[18] Khaine I, Woo SY (2015). An overview of interrelationship between climate change and forests. For. Sci. Technol..

[19] Lagomasino D, Fatoyinbo T, Lee S (2019). Measuring mangrove carbon loss and gain in deltas. Environ. Res. Lett..

[20] Otieno NE, Sajita N, Shitandayi D (2014). Response of a globally endangered canopy insectivore to habitat degradation in an East African tropical rainforest: The role of differential forest protection levels. Int. J. Biodivers. Conserv..

[21] Agevi H (2020). Determination of Species Abundance, Diversity and Carbon Stocks in Kakamega Forest Ecosystem.

[22] Girma A, Fischer E, Dumbo B (2015). Vascular plant diversity and community structure of Nandi forests, western Kenya. J East Afr Nat Hist.

[23] Hicks C, Woroniecki S, Fancourt M, et al. The relationship between biodiversity, carbon storage and the provision of other ecosystem services: Critical Review for the Forestry Component of the International Climate Fund. *Camb U N Environ Programme*. 2014.

[24] Kuria DN, Wachiye SA, Musiega D (2013). GIS based forest cover change and vulnerability analysis: A case study of the Nandi North forest zone. J. Geogr. Reg. Plann..

[25] Vuyiya E, Konje M, Tsingalia H (2014). The impacts of human activities on tree species richness and diversity in Kakamega Forest, Western Kenya. Int. J. Biodivers. Conserv..

[26] Fashing PJ, Nguyen N, Luteshi P (2012). Evaluating the suitability of planted forests for African forest monkeys: A case study from Kakamega Forest, Kenya. Am. J. Primatol..

[27] Martins DJ, Miller SE, Cords M (2015). Observations on an irruption event of the moth Achaea catocaloides (Lepidoptera: Erebidae) at Kakamega Forest, Kenya. J. East Afr. Nat. Hist..

[28] Nyongesah MJ, Li Y (2021). Spatio-temporal variation in species diversity between plantation and secondary Forest of Kakamega tropical rain Forest in Kenya. Ecol. Eng. Environ. Technol..

[29] Mutiso FM, Hitimana J, Kiyiapi JL, et al. Recovery of Kakamega tropical rainforest from anthropogenic disturbances. *J. Trop. For. Sci.* 2013; 566–576.

[30] Tsingalia MH. Regeneration Dynamics of an African Tropical Forest Canopy Dominant Tree Species. In: *Tropical Forests-Ecology, Diversity and Conservation Status*. IntechOpen, 2023.

[31] Statistics KNB of. *The 2019 Kenya Population and Housing Census: Population by County and Sub-county*. Kenya National Bureau of Statistics, 2019.

[32] Melly DK, Kipkoech S, Muema BW (2020). An annotated checklist of the vascular flora of South and North Nandi Forests, Kenya. PhytoKeys.

[33] Agevi WH. Community forest associations and community-based organizations: Redesigning their roles in forest management and conservation in Kenya. 2014.

[34] Mbuvi MTE, Musyoki JK, Ayiemba WO (2015). Determining the potential for introducing and sustaining participatory forest management: A case study of South Nandi Forest of Western Kenya. Int. J. Biodivers. Conserv..

[35] Wabusya M, Pili NN, Bekuta BK (2020). Effects of land-use changes on soil chemical parameters in Kakamega-Nandi Forest Complex. Trop. Subtrop. Agroecosyst..

[36] Chave J, Réjou-Méchain M, Búrquez A (2014). Improved allometric models to estimate the aboveground biomass of tropical trees. Glob. Change Biol..

[37] Chave J, Andalo C, Brown S (2005). Tree allometry and improved estimation of carbon stocks and balance in tropical forests. Oecologia.

[38] Hairiah K, Sitompul SM, Van Noordwijk M, et al. *Methods for sampling carbon stocks above and below ground*. ICRAF Bogor, Indonesia, 2001.

[39] Rozendaal DM, Requena Suarez D, De Sy V (2022). Aboveground forest biomass varies across continents, ecological zones and successional stages: Refined IPCC default values for tropical and subtropical forests. Environ. Res. Lett..

[40] Pellikka PKE, Heikinheimo V, Hietanen J (2018). Impact of land cover change on aboveground carbon stocks in Afromontane landscape in Kenya. Appl. Geogr..

[41] Agevi H. PELIS forestry programme as a strategy for increasing forest cover and improving community livelihoods: case of Malava forest, western Kenya. 2016.

[42] Ameha A, Larsen HO, Lemenih M (2014). Participatory forest management in Ethiopia: Learning from pilot projects. Environ. Manag..

[43] Siraj M, Zhang K, Xiao W (2018). Does participatory forest management save the remnant forest in Ethiopia?. Proc. Natl. Acad. Sci. India Sect. B Biol. Sci..

[44] Feka ZN (2015). Sustainable management of mangrove forests in West Africa: A new policy perspective?. Ocean Coast Manag..

[45] Battles JJ, Bell DM, Kennedy RE, et al. Innovations in measuring and managing forest carbon stocks in California. *Rep California’s Fourth Clim Change Assess* 2018; 99.

[46] Yohannes H, Soromessa T, Argaw M (2015). Carbon stock analysis along forest disturbance gradient in gedo forest: Implications of managing forest for climate change mitigation. J. Ecosyst. Ecography.

[47] Day M, Baldauf C, Rutishauser E (2014). Relationships between tree species diversity and above-ground biomass in Central African rainforests: Implications for REDD. Environ. Conserv..

[48] Kogo BK, Kumar L, Koech R (2019). Forest cover dynamics and underlying driving forces affecting ecosystem services in western Kenya. Remote Sens. Appl. Soc. Environ..

[49] Ali A, Yan E-R, Chen HY (2016). Stand structural diversity rather than species diversity enhances aboveground carbon storage in secondary subtropical forests in Eastern China. Biogeosciences.

[50] Slik JF, Paoli G, McGuire K (2013). Large trees drive forest aboveground biomass variation in moist lowland forests across the tropics. Glob. Ecol. Biogeogr..

[51] Bastin J-F, Fayolle A, Tarelkin Y (2015). Wood specific gravity variations and biomass of central African tree species: The simple choice of the outer wood. PloS One.

[52] Lutz JA, Furniss TJ, Johnson DJ (2018). Global importance of large-diameter trees. Glob. Ecol. Biogeogr..

[53] Nero BF, Callo-Concha D, Denich M (2018). Structure, diversity, and carbon stocks of the tree community of Kumasi, Ghana. Forests.

[54] Kunwar S, Wang L-Q, Chaudhary R (2021). Evolutionary diversity and species richness predict aboveground biomass better than tree size variation in local-scale tropical forest types of Nepal. For. Ecol. Manag..

[55] Li S, Lang X, Liu W (2018). The relationship between species richness and aboveground biomass in a primary Pinus kesiya forest of Yunnan, southwestern China. PloS One.

